# Optimizing and Compensatory Functions of Social Decision-Making Preferences

**DOI:** 10.1093/geroni/igaa057.1833

**Published:** 2020-12-16

**Authors:** Kelly Smith, JoNell Strough, Andrew Parker, Wandi Bruine de Bruin

**Affiliations:** 1 West Virginia University, Morgantown, West Virginia, United States; 2 RAND, Pittsburgh, Pennsylvania, United States; 3 University of Southern California, Los Angeles, California, United States

## Abstract

When making decisions, older people may prefer to work with others to optimize their performance or to compensate for declines in decision-making ability. Using participants from RAND’s American Life Panel (N=1075, Mage = 53.49, we investigated associations among self-reported preferences to make decisions alone and with others, perceived ability to make decisions (compared to age peers and over time), and perceived benefits of aging for decision-making. Older age and perceiving better decision-making abilities relative to peers were associated with greater preferences to make decisions alone and lesser preferences to make decisions with others. Greater preferences for making decisions with others were associated with perceiving improvements in decision-making ability over time and more positive beliefs about aging and decision making. Women were more likely than men to report preferring to make decisions with others. We discuss optimizing and compensatory functions of social preferences for decision making.

